# Assessment of LVEF using a new 16-segment wall motion score in echocardiography

**DOI:** 10.1530/ERP-18-0006

**Published:** 2018-03-21

**Authors:** Real Lebeau, Karim Serri, Maria Di Lorenzo, Claude Sauvé, Van Hoai Viet Le, Vicky Soulières, Malak El-Rayes, Maude Pagé, Chimène Zaïani, Jérôme Garot, Frédéric Poulin

**Affiliations:** 1 Hôpital du Sacré-Coeur de Montréal, Université de Montréal, Montréal, Canada; 2 Institut cardiovasculaire Paris Sud, Massy, France

**Keywords:** left ventricular ejection fraction, transthoracic echocardiography, wall motion score, radionuclide angiography

## Abstract

**Background:**

Simpson biplane method and 3D by transthoracic echocardiography (TTE), radionuclide angiography (RNA) and cardiac magnetic resonance imaging (CMR) are the most accepted techniques for left ventricular ejection fraction (LVEF) assessment. Wall motion score index (WMSI) by TTE is an accepted complement. However, the conversion from WMSI to LVEF is obtained through a regression equation, which may limit its use. In this retrospective study, we aimed to validate a new method to derive LVEF from the wall motion score in 95 patients.

**Methods:**

The new score consisted of attributing a segmental EF to each LV segment based on the wall motion score and averaging all 16 segmental EF into a global LVEF. This segmental EF score was calculated on TTE in 95 patients, and RNA was used as the reference LVEF method. LVEF using the new segmental EF 15-40-65 score on TTE was compared to the reference methods using linear regression and Bland–Altman analyses.

**Results:**

The median LVEF was 45% (interquartile range 32–53%; range from 15 to 65%). Our new segmental EF 15-40-65 score derived on TTE correlated strongly with RNA-LVEF (*r* = 0.97). Overall, the new score resulted in good agreement of LVEF compared to RNA (mean bias 0.61%). The standard deviations (s.d.s) of the distributions of inter-method difference for the comparison of the new score with RNA were 6.2%, indicating good precision.

**Conclusion:**

LVEF assessment using segmental EF derived from the wall motion score applied to each of the 16 LV segments has excellent correlation and agreement with a reference method.

## Introduction

Two-dimensional transthoracic echocardiography (TTE) is a useful non-invasive method for estimating left ventricular (LV) volumes and left ventricular ejection fraction (LVEF). The recommended Simpson biplane method of disks has intrinsic limitations such as its dependence on geometric assumptions and the limited number of views used to derive global LVEF (1). Radionuclide angiography is a reference method with less operator dependency and excellent interobserver variability but involves exposure to ionizing radiation (2). Cardiac magnetic resonance imaging (CMR) is the gold standard for the precise estimation of LV volumes and LVEF requiring meticulous tracings of endocardial borders in short-axis planes (3). Using TTE, wall motion score index (WMSI) can be derived and used as an alternative for evaluating LVEF (1). However, the conversion from a WMSI to LVEF is obtained through a regression equation, which may limit its routine use. In this retrospective study, we aimed to validate a new method to derive LVEF from the wall motion score in 95 patients undergoing TTE.

## Methods

### Patient selection

We included 95 randomly selected patients who underwent both TTE and radionuclide angiography (RNA). Patients with poor diagnostic quality of echocardiographic images or LVEF greater than 65% (by RNA) were excluded. Furthermore, patients with severe valvular disease, hypertrophic cardiomyopathy or congenital heart disease were also excluded. Consent has been obtained from each patient after full explanation of the purpose and nature of all procedures used. The study protocol was approved by the Hôpital du Sacré-Coeur de Montréal Research Ethics Board.

### Echocardiography

**Figure 1:**
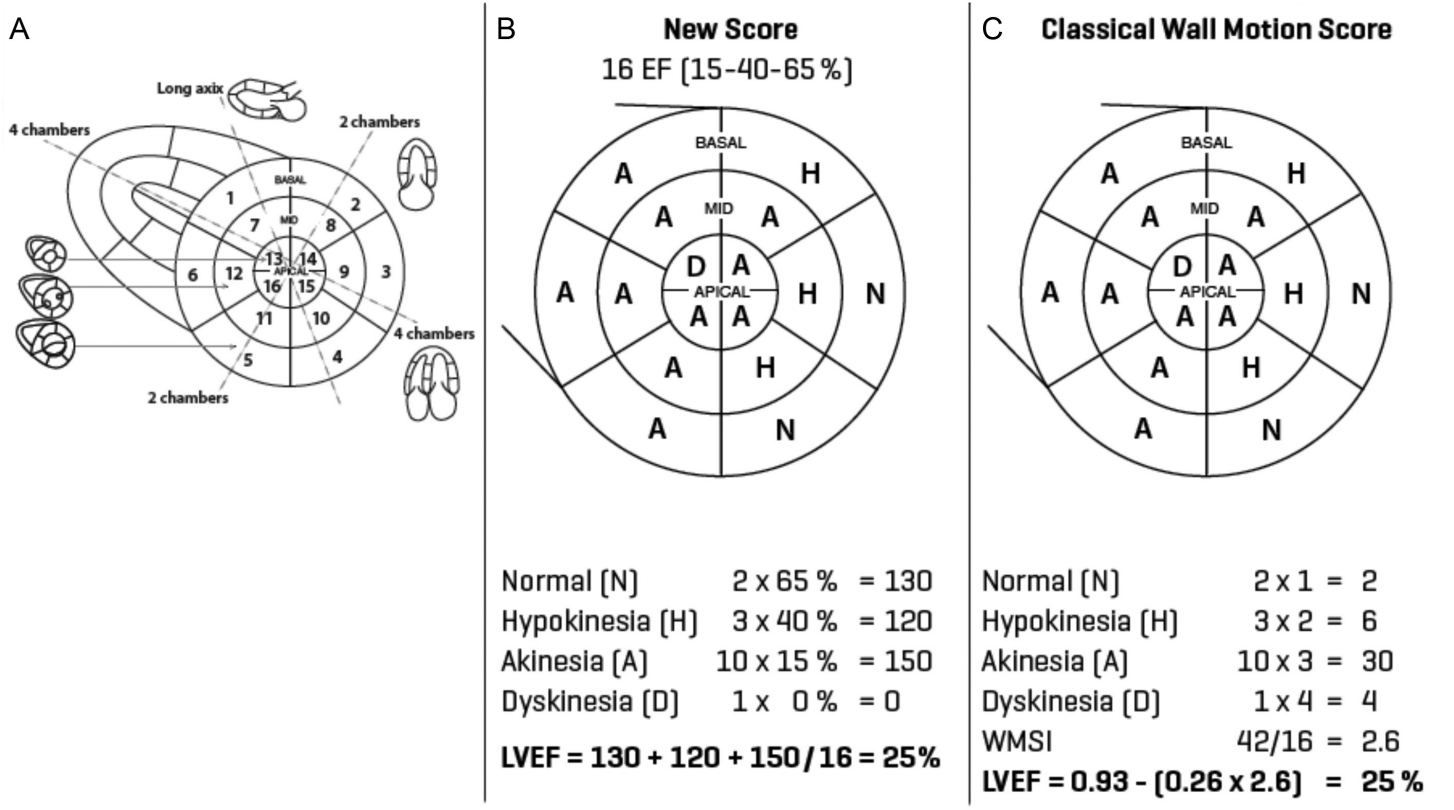
Bull’s eye representation of the sixteen-segment model as suggested by the American Society of Echocardiography (A) accompanied by an example of calculation of the new segmental EF 15-40-65 score (B) and the classical WMSI (C) in a patient with severe ischemic cardiomyopathy. LVEF, left ventricular ejection fraction; WMSI, wall motion score index.

Transthoracic echocardiograms and Doppler studies were performed in accordance with the American Society of Echocardiography (ASE) guidelines. All standard parasternal long- and short-axis as well as apical views were used to qualitatively derive the classical wall motion score (1). RNA and TTE were performed within three days of one another. The technique used to perform RNA was detailed previously (4). Visual semi-quantitative assessment of regional wall motion and thickening for wall motion score was performed by an experienced cardiologist in a blinded fashion. Wall motion score was transposed to a graphic representation of the cardiac polar map (Fig. 1). WMSI was also converted to LVEF through a validated regression equation (LVEF = 0.93 − (0.26 × WMSI)) (Table 1) (4).

**Table 1:** The conversion of echo and CMR WMSI into LVEF by regression models in three studies of 1132 patients.

WMSI	Reference LVEF methods			
	RNA-LVEF^*^ (*N* = 243) (4)	Semi-quantitative^†^ echo LVEF^*^ (*N* = 767) (8)	CMR LVEF^‡^ (*N* = 122) (9)	
1.0	67	64	64	Normokinesia
1.1	65	62	62	
1.2	62	59	59	
1.3	61	56	56	
1.4	57	54	54	
1.5	54	51	51	Mild hypokinesia
1.6	53	48	48	
1.7	50	46	46	
1.8	47	43	43	
1.9	44	41	41	
2.0	41	38	38	Hypokinesia
2.1	39	35	35	
2.2	36	33	33	
2.3	34	30	30	
2.4	31	28	28	
2.5	28	25	25	Severe hypokinesia
2.6	26	22	22	
2.7	24	20	20	
2.8	21	17	17	
2.9	18	14	14	
3.0	15	12	12	Akinesia

^*^WMSI calculated using the transthoracic echocardiogram; ^†^Semi-quantitative echo LVEF was defined as the combination of the modified method of Quinones combined with an overall visual estimation of LVEF; ^‡^WMSI calculated using the cardiac magnetic resonance.

### New segmental EF 15-40-65 score

The new score consisted of attributing a segmental ejection fraction (EF) to each of the LV segments based on the wall motion score and averaging all 16 segmental EFs into a global LVEF. It was derived from the standard regional wall motion assessment using a 16-segment LV model (6 basal, 6 mid and 4 apical segments) as recommended by the ASE (1). The new score relies on a correspondence between a segmental wall motion score and a segmental EF defined as follows: normokinesia (EF = 65%), hypokinesia (EF = 40%) and akinesia (EF = 15%), corresponding respectively to normal motion and normal thickening, reduced motion and thickening and absence of motion and thickening of the segment. Hypokinesia was further subdivided into mild hypokinesia (EF = 50%) and severe hypokinesia (EF = 30%). Dyskinetic (systolic bulging) and aneurysmal (systolic and diastolic bulging deformation) segments were attributed EF = 0%. In this simplified score, hyperkinesia was considered equivalent to normokinesia. The global LVEF was obtained by averaging all 16 segmental EF into a global LVEF (Fig. 1).

#### Intraobserver and interobserver variability

Twenty randomly selected studies were reanalyzed by the same operator several months after the initial analysis. A second experienced observer, also blinded to previously obtained data, analyzed the same loops for the assessment of interobserver variability of the wall motion score.

### Statistical analysis

Categorical variables are expressed in frequency and percentages. Normality of distribution of continuous variables is assessed with the Shapiro–Wilk test. Continuous variables are described as mean ± standard deviation (s.d.) if the distribution was normal, otherwise as median and interquartile range (IQR) (25th–75th). LVEF derived using the new segmental EF 15-40-65 score was compared with the reference method using linear regression and Bland–Altman analysis.

Using Bland–Altman analysis, we evaluated systematic bias (using mean differences between methods), s.d. of inter-method difference and precision (range within which are 95 of values of differences between methods, i.e. ±1.96 s.d. of differences between methods) (5). The intraobserver and interobserver variability were evaluated with the intraclass correlation coefficient. All statistical analyses were conducted using SPSS, version 24 (SPSS). When applicable, a two-tailed *P* value of 0.05 was used for all analysis.

## Results

Our cohort consisted of 95 individuals (mean age 69, range 21–88 years, 42% female) with a median RNA-LVEF of 45% (IQR 30–59%; range from 15 to 65%). Ischemic cardiomyopathy (*n* = 72; 75%) was the most common diagnosis followed by dilated cardiomyopathy (*n* = 14; 15%).

### Validation of the new segmental EF 15-40-65 score

**Figure 2:**
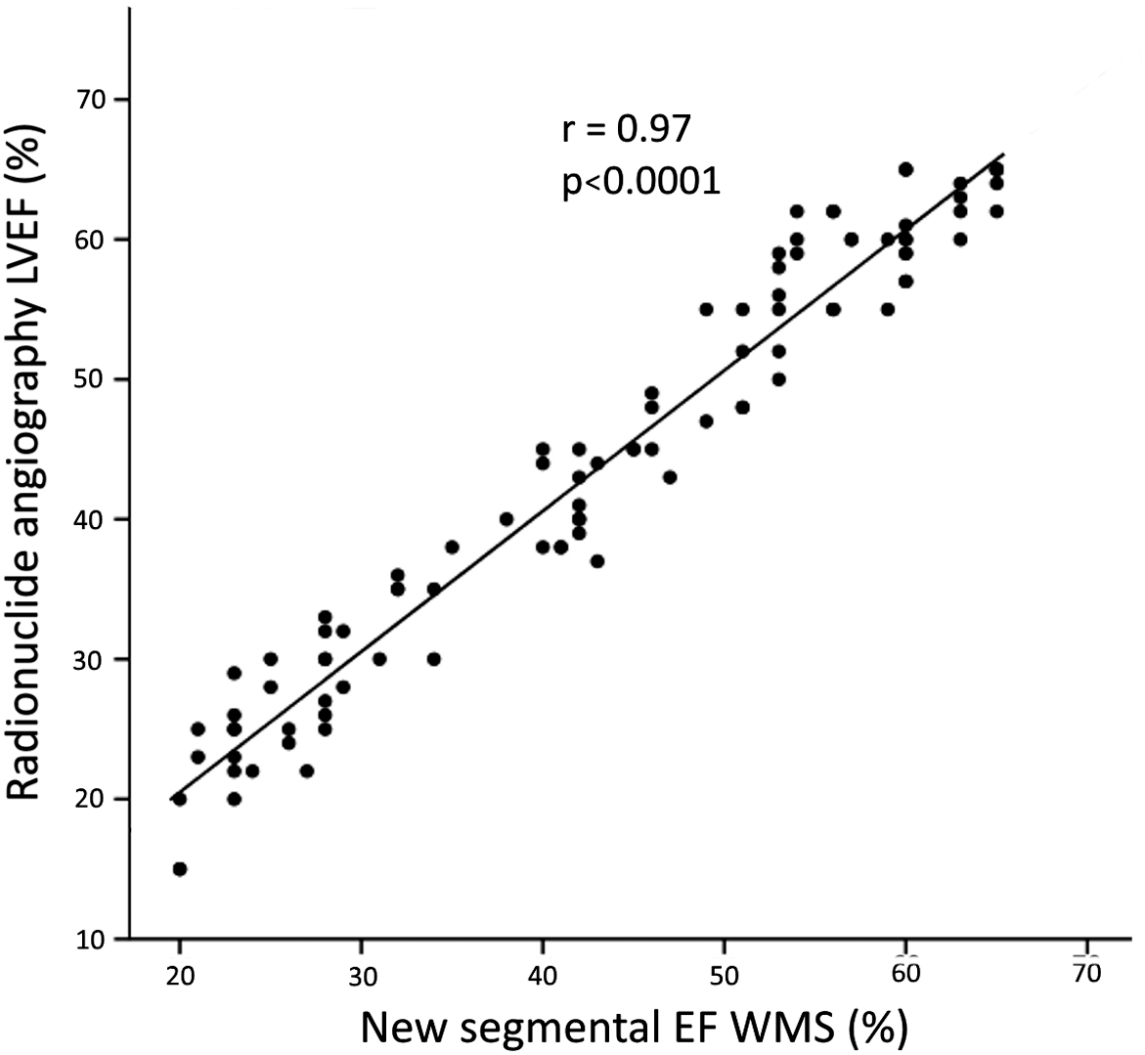
Comparison between the new segmental EF 15-40-65 score and radionuclide angiography. Linear regression analysis between the new segmental EF 15-40-65 score vs RNA-LVEF. LVEF, left ventricular ejection fraction; RNA, radionuclide angiography.

The median LVEF obtained by the new segmental EF 15-40-65 score was 43% (IQR 28–56; range from 20 to 65%). The correlation between RNA-LVEF and the new segmental EF 15-40-65 score was excellent (*R* = 0.97) (Fig. 2).

### Analysis of systematic bias

**Figure 3:**
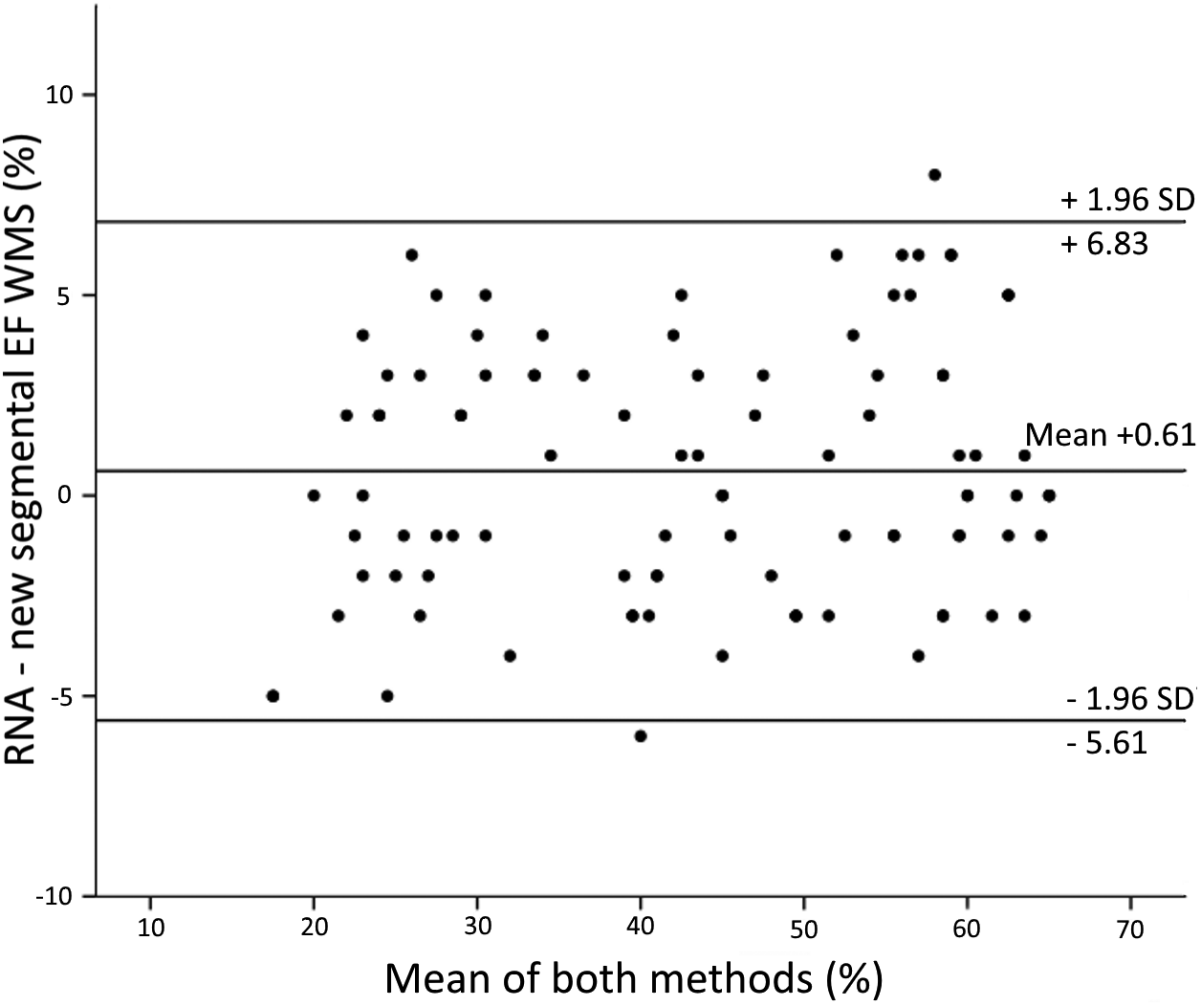
Comparison between the new segmental EF 15-40-65 score and RNA-LVEF. Bland–Altman analysis of the new segmental EF 15-40-65 score vs RNA-LVEF. LVEF, left ventricular ejection fraction; RNA, radionuclide angiography.

Bland–Altman analysis showed excellent agreement between the new score and RNA (mean LVEF bias = 0.61%) (Fig. 3 and Table 2). Linear regression analysis confirmed the absence of proportional bias.

**Table 2:** Comparison between RNA-LVEF and LVEF derived from new segmental EF 15-40-65 score on TTE.

	Mean difference between methods ± s.d. (%)	Precision (%)
RNA-LVEF vs segmental EF 15-40-65 score on TTE	0.61 ± 3.17	12.4

LVEF, left ventricular ejection fraction; RNA, radionuclide angiography.

### Analysis of precision

The s.d. of the distribution of inter-method differences between the new score and RNA-LVEF were acceptable. The s.d. was ±3.17% (±7% of the median RNA-LVEF). Consequently, the 95% confidence interval of inter-method difference (±1.96 s.d. i.e. precision) was 12.4% (Fig. 3 and Table 2).

Assessment of LVEF using the regression-based method from the classic wall motion score was not superior to the new segmental EF 15-40-65 score showing equivalent correlation vs RNA-LVEF (*R* = 0.97) and similar accuracy and precision (Supplementary Table, see section on supplementary data given at the end of this article).

### Reproducibility

Analysis of intraobserver and interobserver variability for the wall motion score demonstrated good agreement between observations (intraobserver 0.97; interobserver 0.97).

## Discussion

In this study of patients with a broad range of LVEF referred for TTE, we aimed to test the validity of a simplified method to convert segmental wall motion assessment into segmental EF to easily obtain the global LVEF. The main results were:

1. The use of the new segmental EF (15-40-65) score resulted in excellent correlation and excellent systematic bias overall compared with reference LVEF methods.
2. Precision of this new score by echocardiography to assess LVEF was clinically acceptable compared with a reference method.

### Classical WMSI

The WMSI has been previously validated in several echocardiographic studies and well correlated with RNA-LVEF (6, 7). We published in 2003 an assessment of LVEF derived from WMSI in 243 patients using the classical 16-segment model, as recommended by the ASE (4). We obtained an excellent correlation (*r* = 0.88) and agreement with RNA-LVEF in all stratum of LVEF. The regression equation derived was RNA EF = 0.93 − (0.26 × WMSI), which has been reproduced thereafter by Moller *et al.* from the Mayo Clinic using TTE (8) and by Lebeau *et al.* on CMR (9) in a total of 1132 patients (Table 1). In a study of 110 patients by Duncan *et al.* comparing echocardiographic LVEF derived from the WMSI, echocardiographic Simpson’s biplane method and LVEF obtained by CMR, WMSI-derived LVEF had a stronger correlation with CMR than Simpson’s biplane method and was more accurate (10). In a systematic review by McGowan *et al.* WMSI-LVEF has proven to be a valid method to assess LVEF (11), potentially superior to Simpson’s biplane method. Similarly, in a CMR study involving 203 patients, Sierra-Galan *et al.* have demonstrated that the WMSI is an accurate method to measure LVEF (12) and is a useful complement to automatic border detection that can often be erratic.

We believe that the WMSI model has several advantages over the planimetric method. The WMSI method takes advantage of the multiple incidences of the LV to complete a polar map as opposed to only two sagittal views used for the classical Simpson’s biplane method. The Simpson formula is also less precise in patients with very abnormal ventricular geometry (dyskinesia or aneurysm) (1). In the setting of acute myocardial infarction, WMSI reflects the size of myocardial infarction better than LVEF (13, 14, 15). WMSI is also superior to LVEF for risk stratification (death and re-hospitalization for heart failure, re-MI, stoke) after acute myocardial infarction (8, 16, 17, 18, 19).

### Simplified wall motion score

We have previously shown that a semi-quantitative method dedicated specifically to emergency and critical care physicians based on the simple assessment of normokinesia (EF = 60%), hypokinesia (EF = 40%) or akinesia (EF = 20%) in the 3 short-axis views was superior to global visual estimation (20, 21). In the current study, we applied the new segmental EF 15-40-65 score in TTE to each of the 16 segments of the polar map and obtained a global LVEF resulting from the arithmetic mean of all segmental EF. The rationale behind this segmental EF using WMS is the strong linear relation between WMSI and LVEF (4, 8, 9). Indeed, in previous studies of global LVEF, the global WMSI was translated into LVEF by a mathematical regression model (Table 1) (4, 8, 9). For instance, a normal LV function with no wall motion abnormalities has a score of 1.0 corresponding to an LVEF of 67% and CMR LVEF of 64% (65% was used as mean approximation). Mild diffuse hypokinesia with a score of 1.5 has an echo LVEF of 54% and CMR LVEF of 51% (mean approximation = 50%). Diffuse hypokinesia with a score of 2 has an echo LVEF of 41% and CMR LVEF of 38% (mean approximation = 40%). Severe diffuse hypokinesia with a score of 2.5 has an echo LVEF of 28% and CMR LVEF of 25% (mean approximation = 30%). Diffuse akinesia has an echo LVEF of 15% and CMR LVEF of 12% (mean approximation = 15%). We simply assigned a segmental EF for each segment based on the aforementioned convention, translating segmental wall motion score into a segmental EF and then obtained global LVEF by averaging the 16-segment EF.

This new method to derive global LVEF was accurate compared to a reference method. We consider that the precision obtained (12%) with this new segmental EF 15-40-65 score was reasonable. Indeed, it compares favorably to the precision of three-dimensional echocardiography compared with CMR in a meta-analysis (precision of 3D = 24%) (22) and in the more recent study of Hoffmann *et al.* (precision of 3D = 48%) (23). Thus, the use of this simple and highly reproducible method to easily obtain global LVEF from the bull’s eye representation of wall motion score using all standard views, which has not been described before, has the potential to refine the daily assessment of systolic function in busy echocardiography laboratories and at the bedside by sonographers and cardiologists.

### Perspectives of wall motion score

It is becoming accepted that strain and 3D have superior sensitivity and reproducibility to detect LV systolic dysfunction (1). However, they require more expertise and several technical pitfalls and standardization issues remain. With the widespread utilization of portable and handheld echocardiography in emergency departments and intensive care units to evaluate patient hemodynamics, LV wall motion assessment is an easier method to assess LV systolic function and is generalizable to a wider range of physicians with various level of training, expertise and echocardiographic equipment (24, 25, 26). We have shown herein that a simple segmental score (EF 15-40-65) applied to each of the 16 segments results in a robust estimation of LVEF by echocardiography.

### Limitations

The most significant limitation remains the capacity to obtain good images and sufficient ability to interpret motion abnormalities. The wall motion scores were assessed by an experienced echocardiographer. While we acknowledge that estimation of the wall motion abnormalities can be subjective, our interobserver variability was very acceptable. In cases where there is discrepant wall motion score in a given segment, we believe that short-axis parasternal views should be weighted more heavily in the final score of that segment. Indeed, short-axis views offer the only real 3D (360°) analysis of cardiac dynamics as opposed to the limited degrees of evaluation obtained with the thin sagittal cuts from the apical views.

This model of wall motion score is not designed for the evaluation of hyperkinetic states, because the maximal score by our semi-quantitative model is 65%. This model should be reserved for patients with LV dysfunction and used cautiously in patient with localized hyperkinetic wall motion since it will result in an underestimation of LVEF. Whether this new score can be extrapolated to a 17-segment model is likely but remains to be validated. The apical cap is a small segment with little intrinsic kinesis and is more useful to assess in perfusion studies. Additionally, attributing a segmental EF score of 0% to dyskinetic or aneurysmal segments was preferred to a negative correction (for example, segmental EF of −15%) since the latter would have led to systematic underestimation of LVEF in exploratory analyses of 11 patients with such segmental abnormalities. Finally, improving endocardial definition in TTE with the use of contrast agents could have resulted in improved precision of the new segmental EF 15-40-65 score (27).

## Conclusion

Our results suggest that the estimation of LVEF using a new segmental EF 15-40-65 score is an accurate method and a good complement to planimetric methods. Any echocardiography laboratory that systematically assesses LV function by grading the wall motion score (16-segment model) can then easily translate this routine information into a robust estimate of LVEF without the use of regression equations.

## Supplementary Material

Supplemental Table 1. Comparison between RNA-LVEF and LVEF derived from the classic wall motion score index using the regression equation

## Declaration of interest

The authors declare that there is no conflict of interest that could be perceived as prejudicing the impartiality of the research reported.

## Funding

This research did not receive any specific grant from any funding agency in the public, commercial or not-for-profit sector.

## References

[1] Lang RM Badano LP Mor-Avi V Afilalo J Armstrong A Ernande L Flachskampf FA Foster E Goldstein SA Kuznetsova T, ***et al*** Recommendations for cardiac chamber quantification by echocardiography in adults: an update from the American Society of Echocardiography and the European Association of Cardiovascular Imaging. Journal of the American Society of Echocardiography 2015 28 1.e14–39.e14. (10.1016/j.echo.2014.10.003) 25559473

[2] Corbett JR Akinboboye OO Bacharach SL Borer JS Botvinick EH DePuey EG Ficaro EP Hansen CL Henzlova MJ Van Kriekinge S, ***et al*** Equilibrium radionuclide angiocardiography. Journal of Nuclear Cardiology 2006 13 e56–e79. (10.1016/j.nuclcard.2006.08.007) 17174797

[3] Bellenger NG Davies LC Francis JM Coats AJ Pennell DJ. Reduction in sample size for studies of remodeling in heart failure by the use of cardiovascular magnetic resonance. Journal of Cardiovascular Magnetic Resonance 2000 2 271–278. (10.3109/10976640009148691) 11545126

[4] Lebeau R Di Lorenzo M Amyot R Veilleux M Lemieux R Sauve C. A new tool for estimating left ventricular ejection fraction derived from wall motion score index. Canadian Journal of Cardiology 2003 19 397–404. 12704486

[5] Hanneman SK. Design, analysis, and interpretation of method-comparison studies. AACN Advanced Critical Care 2008 19 223–234. 18560291 10.1097/01.AACN.0000318125.41512.a3 PMC2944826

[6] Berning J Rokkedal Nielsen J Launbjerg J Fogh J Mickley H Andersen PE. Rapid estimation of left ventricular ejection fraction in acute myocardial infarction by echocardiographic wall motion analysis. Cardiology 1992 80 257–266. (10.1159/000175011) 1511472

[7] Rifkin RD Koito H. Comparison with radionuclide angiography of two new geometric and four nongeometric models for echocardiographic estimation of left ventricular ejection fraction using segmental wall motion scoring. American Journal of Cardiology 1990 65 1485–1490. (10.1016/0002-9149(90)91360-I) 2353656

[8] Moller JE Hillis GS Oh JK Reeder GS Gersh BJ Pellikka PA. Wall motion score index and ejection fraction for risk stratification after acute myocardial infarction. American Heart Journal 2006 151 419–425. (10.1016/j.ahj.2005.03.042) 16442909

[9] Lebeau R Serri K Morice MC Hovasse T Unterseeh T Piechaud JF Garot J. Assessment of left ventricular ejection fraction using the wall motion score index in cardiac magnetic resonance imaging. Archives of Cardiovascular Diseases 2012 105 91–98. (10.1016/j.acvd.2012.01.002) 22424327

[10] Duncan RF Dundon BK Nelson AJ Pemberton J Williams K Worthley MI Zaman A Thomas H Worthley SG. A study of the 16-Segment Regional Wall Motion Scoring Index and biplane Simpson's rule for the calculation of left ventricular ejection fraction: a comparison with cardiac magnetic resonance imaging. Echocardiography 2011 28 597–604. (10.1111/j.1540-8175.2011.01394.x) 21718352

[11] McGowan JH Cleland JG. Reliability of reporting left ventricular systolic function by echocardiography: a systematic review of 3 methods. American Heart Journal 2003 146 388–397. 12947354 10.1016/S0002-8703(03)00248-5

[12] Sierra-Galan LM Ingkanisorn WP Rhoads KL Agyeman KO Arai AE. Qualitative assessment of regional left ventricular function can predict MRI or radionuclide ejection fraction: an objective alternative to eyeball estimates. Journal of Cardiovascular Magnetic Resonance 2003 5 451–463. (10.1081/JCMR-120022261) 12882076

[13] Baron T Flachskampf FA Johansson K Hedin EM Christersson C. Usefulness of traditional echocardiographic parameters in assessment of left ventricular function in patients with normal ejection fraction early after acute myocardial infarction: results from a large consecutive cohort. European Heart Journal: Cardiovascular Imaging 2016 17 413–420. (10.1093/ehjci/jev160) 26139362

[14] Munk K Andersen NH Nielsen SS Bibby BM Botker HE Nielsen TT Poulsen SH. Global longitudinal strain by speckle tracking for infarct size estimation. European Journal of Echocardiography 2011 12 156–165. (10.1093/ejechocard/jeq168) 21131657

[15] Eek C Grenne B Brunvand H Aakhus S Endresen K Hol PK Smith HJ Smiseth OA Edvardsen T Skulstad H. Strain echocardiography and wall motion score index predicts final infarct size in patients with non-ST-segment-elevation myocardial infarction. Circulation: Cardiovascular Imaging 2010 3 187–194. (10.1161/CIRCIMAGING.109.910521) 20075142

[16] Munk K Andersen NH Terkelsen CJ Bibby BM Johnsen SP Botker HE Nielsen TT Poulsen SH. Global left ventricular longitudinal systolic strain for early risk assessment in patients with acute myocardial infarction treated with primary percutaneous intervention. Journal of the American Society of Echocardiography 2012 25 644–651. (10.1016/j.echo.2012.02.003) 22406163

[17] Galasko GI Basu S Lahiri A Senior R. A prospective comparison of echocardiographic wall motion score index and radionuclide ejection fraction in predicting outcome following acute myocardial infarction. Heart 2001 86 271–276. (10.1136/heart.86.3.271) 11514477 PMC1729882

[18] Jurado-Roman A Agudo-Quilez P Rubio-Alonso B Molina J Diaz B Garcia-Tejada J Martin R Tello R. Superiority of wall motion score index over left ventricle ejection fraction in predicting cardiovascular events after an acute myocardial infarction. European Heart Journal: Acute Cardiovascular Care 2016 [epub]. (10.1177/2048872616674464) 27738092

[19] Carluccio E Tommasi S Bentivoglio M Buccolieri M Prosciutti L Corea L. Usefulness of the severity and extent of wall motion abnormalities as prognostic markers of an adverse outcome after a first myocardial infarction treated with thrombolytic therapy. American Journal of Cardiology 2000 85 411–415. (10.1016/S0002-9149(99)00764-X) 10728942

[20] Lebeau R Sas G El Rayes M Serban A Moustafa S Essadiqi B DiLorenzo M Souliere V Beaulieu Y Sauve C, ***et al*** Left ventricular ejection fraction assessment by non-cardiologists from transverse views using a simplified wall motion score index. Echo Research and Practice 2015 2 1–8. (10.1530/ERP-14-0003) 26693310 PMC4676426

[21] Lebeau R Potter B Sas G Moustafa S di Lorenzo M Souliere V Beaulieu Y Sauve C Amyot R Serri K. Performance of a simplified wall motion score index method for noncardiologists to assess left ventricular ejection fraction. ISRN Emergency Medicine 2012 2012 article ID 309470. (10.5402/2012/309470)

[22] Dorosz JL Lezotte DC Weitzenkamp DA Allen LA Salcedo EE. Performance of 3-dimensional echocardiography in measuring left ventricular volumes and ejection fraction: a systematic review and meta-analysis. JACC: Journal of the American College of Cardiology 2012 59 1799–1808. (10.1016/j.jacc.2012.01.037) 22575319 PMC3773600

[23] Hoffmann R Barletta G von Bardeleben S Vanoverschelde JL Kasprzak J Greis C Becher H. Analysis of left ventricular volumes and function: a multicenter comparison of cardiac magnetic resonance imaging, cine ventriculography, and unenhanced and contrast-enhanced two-dimensional and three-dimensional echocardiography. Journal of the American Society of Echocardiography 2014 27 292–301. (10.1016/j.echo.2013.12.005) 24440110

[24] Labovitz AJ Noble VE Bierig M Goldstein SA Jones R Kort S Porter TR Spencer KT Tayal VS Wei K. Focused cardiac ultrasound in the emergent setting: a consensus statement of the American Society of Echocardiography and American College of Emergency Physicians. Journal of the American Society of Echocardiography 2010 23 1225–1230. (10.1016/j.echo.2010.10.005) 21111923

[25] Farsi D Hajsadeghi S Hajighanbari MJ Mofidi M Hafezimoghadam P Rezai M Mahshidfar B Abiri S Abbasi S. Focused cardiac ultrasound (FOCUS) by emergency medicine residents in patients with suspected cardiovascular diseases. Journal of Ultrasound 2017 20 133–138. (10.1007/s40477-017-0246-5) 28593003 PMC5440337

[26] Heiberg J El-Ansary D Canty DJ Royse AG Royse CF. Focused echocardiography: a systematic review of diagnostic and clinical decision-making in anaesthesia and critical care. Anaesthesia 2016 71 1091–1100. (10.1111/anae.13525) 27346556

[27] Mulvagh SL Rakowski H Vannan MA Abdelmoneim SS Becher H Bierig SM Burns PN Castello R Coon PD Hagen ME, ***et al*** American Society of Echocardiography Consensus statement on the clinical applications of ultrasonic contrast agents in echocardiography. Journal of the American Society of Echocardiography 2008 21 1179–1201. (10.1016/j.echo.2008.09.009) 18992671

