# Supplementary material for: Assessment of LVEF using a new 16-segment wall motion score in echocardiography
Source: Echo Res Pract. 2018 Mar 21;5(2):63–9. doi: 10.1530/ERP-18-0006 (PMC5887066; doi:10.1530/ERP-18-0006)
Supplement: Supplemental Table 1. [file erp-5-63-t001.pdf]

Supplemental Table 1. Comparison between RNA-LVEF and LVEF derived from the classic wall motion score index using the regression equation

	**Mean difference between methods ± SD, %**	**Precision, %**
RNA LVEF vs. regression-based WMS method LVEF on TTE	0.38 ± 3.06	12.0

RNA, Radionuclide angiography; LVEF, left ventricular ejection fraction, WMS, wall motion score
